# Autonomic and Redox Imbalance Correlates With T-Lymphocyte Inflammation in a Model of Chronic Social Defeat Stress

**DOI:** 10.3389/fnbeh.2019.00103

**Published:** 2019-05-14

**Authors:** Cassandra M. Moshfegh, Safwan K. Elkhatib, Christopher W. Collins, Allison J. Kohl, Adam J. Case

**Affiliations:** Department of Cellular and Integrative Physiology, University of Nebraska Medical Center, Omaha, NE, United States

**Keywords:** post-traumatic stress disorder, PTSD, behavior, norepinephrine, immune, IL-6, IL-17, calprotectin

## Abstract

Patients diagnosed with post-traumatic stress disorder (PTSD) are at a significantly elevated risk of developing comorbid inflammatory conditions, but the mechanisms underlying this predilection remain unclear. Our previous work has shown that T-lymphocytes exposed to elevated levels of norepinephrine (NE) displayed a pro-inflammatory signature reminiscent of an autoreactive phenotype. With this, we hypothesized that the increased sympathetic tone observed during psychological trauma may be promoting pro-inflammatory T-lymphocytes, which causes a predisposition to comorbid inflammatory conditions. Here, we examined the consequences of psychological trauma on splenic T-lymphocytes using a mouse model of repeated social defeat stress. Social defeat led to anxiety-like and depression-like behavior as has been previously described. The spleens of socially-defeated mice showed significant elevations of NE, tyrosine hydroxylase (TH), and acetylcholinesterase (ACHE) levels, which appeared to be due in part to increased expression within T-lymphocytes. Additionally, T-lymphocytes from stressed animals showed higher levels of pro-inflammatory cytokines and mitochondrial superoxide. Interestingly, in this model system, close associations exist within splenic T-lymphocytes amid the autonomic, inflammatory, and redox environments, but these only weakly correlate with individual behavioral differences among animals suggesting the psychological and physiological manifestations of trauma may not be tightly coupled. Last, we describe, for the first time, elevations in calprotectin levels within T-lymphocytes and in circulation of psychologically stressed animals. Calprotectin correlated with both behavioral and physiological changes after social defeat, suggesting the potential for a new biological marker and/or therapeutic target for psychological trauma and its inflammatory comorbidities.

## Introduction

Approximately 70% of adults in the United States have experienced some form of traumatic event, and development of post-traumatic stress disorder (PTSD) in this population is estimated at over 20% or 45 million Americans (Kessler et al., 1995, 2005, 2012). PTSD is classified as a trauma and stressor-related disorder, and the disease manifests itself in several behavioral changes including intrusion symptoms, avoidance, and negative alterations in cognitions and mood (American Psychiatric Association, 2013). PTSD patients also demonstrate significantly elevated risks for the development of comorbid somatic illnesses such as cardiovascular, metabolic, and autoimmune diseases (Boscarino, 2004; Edmondson et al., 2013; Mikuls et al., 2013; Britvić et al., 2015; Lee et al., 2016; Edmondson and von Känel, 2017). While PTSD patients frequently partake in activities that independently increase the chances of developing these disorders (e.g., smoking, drug use, poor diet, lack of exercise, etc.), statistical analyses have explicitly shown a consistent and significantly elevated comorbid disease risk even after controlling for these precarious activities. Moreover, it is unclear if treatment of the behavioral manifestations of PTSD impact the development of these comorbid somatic conditions, suggesting the control mechanisms remain elusive.

One characteristic physiological change of PTSD that may partially explain the development of comorbid somatic diseases is elevated sympathetic nervous system activity and norepinephrine (NE) outflow (Park et al., 2017). Compared to other psychological conditions such as chronic depression, bipolar, or schizophrenia disorders, PTSD patients show significantly elevated NE levels in both urine and cerebrospinal fluid compared to matched controls (Mason et al., 1988; Geracioti et al., 2001, 2008; Strawn et al., 2004). Moreover, targeting of NE *via* pharmacological means using prazosin or clonidine (α1 adrenergic antagonist and α2 adrenergic agonist, respectively), physical manipulation by denervation of the sympathetic chain, as well as anesthetic ganglion blockade have all demonstrated benefits in attenuating the psychological manifestations of the disease (Sutherland and Davidson, 1994; Brady et al., 2000; Telaranta, 2003; Raskind et al., 2007; Lipov et al., 2008, 2012; Lipov and Kelzenberg, 2012). These treatment modalities are highly suggestive of a sympathetic component contributing to PTSD, however, it remains unclear if this dysregulation of autonomic tone is causal to the development of comorbid somatic diseases.

Inflammation is also a theme of all the comorbid diseases described in PTSD to date, and the immune system, particularly T-lymphocytes, appear to be highly sensitive to the psychobiological and sympathetic changes after trauma. For example, PTSD patients have decreased numbers of naïve and regulatory (anti-inflammatory) T-lymphocytes with concurrent increases in memory T-lymphocytes (Sommershof et al., 2009; Wilson et al., 2012). Additionally, circulating levels of various pro-inflammatory cytokines such as interleukin 6 (IL-6) and interleukin 17A (IL-17A) have been shown to be elevated in the PTSD population (von Känel et al., 2007; Zhou et al., 2014; Imai et al., 2018; Maloley et al., 2019). Animal models have corroborated these results showing alterations in both T-lymphocyte populations and cytokine production with various modalities of traumatic stress induction (Avitsur et al., 2002; Hodes et al., 2014). We and others have previously demonstrated that exposure to simply elevated levels of NE can have profound effects on T-lymphocyte activation and cytokine production (Padro and Sanders, 2014; Case and Zimmerman, 2015; Case et al., 2016), and our recent report has elucidated a novel role for the mitochondrial redox environment in NE-mediated T-lymphocyte regulation (Case et al., 2016). Taken together along with the observation that glucocorticoid levels are often not elevated in patients with PTSD (Mason et al., 1988), we hypothesized that the increased sympathoexcitation observed in PTSD is leading to an increased pro-inflammatory T-lymphocyte phenotype *via* redox mechanisms, and it is this inflammation that predisposes these patients to increased incidences of comorbid somatic diseases.

To address this hypothesis, herein, we utilized an established and accepted mouse model of psychological trauma known as repeated social defeat (Golden et al., 2011; Deslauriers et al., 2018). We show that these animals demonstrated altered behavior, dysregulated autonomic balance with elevated sympathetic tone, and increased T-lymphocyte pro-inflammatory cytokine production concurrent with a disrupted mitochondrial redox environment, which confirms and extends our previous observations using *in vitro* systems (Case et al., 2016). However, examination of individual animal differences identified that only a few physiological parameters associated significantly with specific behavioral phenotypes, but are highly related to other respective physiological elements. Last, T-lymphocyte RNA sequencing identified expression of a novel and unexpected inflammatory protein (i.e., calprotectin) within these cells from stressed animals that correlates with both behavioral phenotypes and physiological readouts, suggesting the potential for a new biomarker and/or regulatory player of psychological trauma.

## Materials and Methods

### Mice

All control and experimental stress animals were 8-12 week-old male wild-type mice of a C57BL/6J background (Jackson Laboratory #000664, Bar Harbor, ME, USA). The social defeat stress paradigm precludes the use of female mice, thus, sex differences were not examined and not within the scope of the study described herein. All aggressive mice were 4-6 month-old retired breeder male mice of a CD-1 background (Charles River #022, Wilmington, MA, USA). Experimental mice were bred in-house to eliminate shipping stress and environmental changes. Littermates were group housed (≤5 mice per cage) prior to the stress induction protocol to eliminate social isolation stress. Mice were housed with standard corncob bedding, paper nesting material, and given access to standard chow (Teklad Laboratory Diet #7012, Harlan Laboratories, Madison, WI, USA) and water *ad libitum*. Mice were euthanized by pentobarbital overdose (150 mg/kg, Fatal Plus, Vortech Pharmaceuticals, Dearborn, MI, USA) administered intraperitoneally. All mice were sacrificed between 07:00 and 09:00 Central Standard Time to eliminate circadian rhythm effects on T-lymphocyte function. Mice were randomized prior to the start of all experiments, and when possible, experimenters were blinded to the control and stress groups of mice until the completion of the study. All procedures were reviewed and approved by the University of Nebraska Medical Center Institutional Animal Care and Use Committee.

### Social Defeat Stress Paradigm

**Figure 1:**
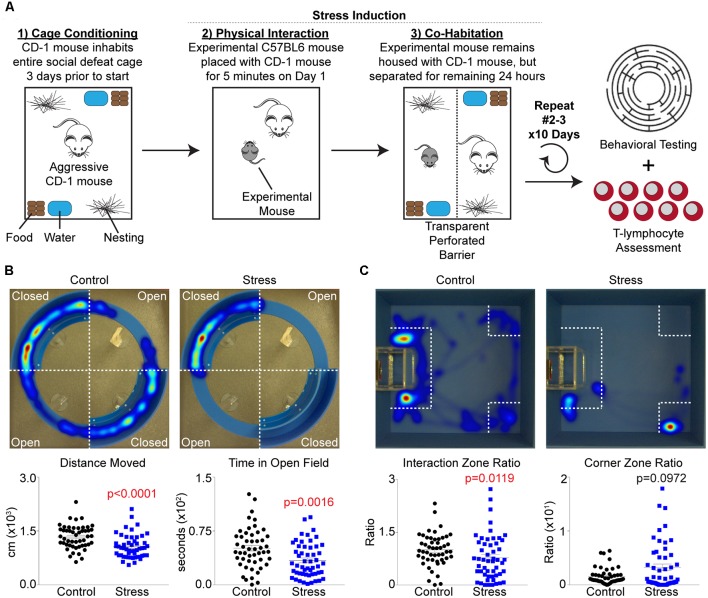
Repeated social defeat stress increases anxiety-like and depression-like behavior. **(A)** Overview of experimental setup and timeline. **(B)** *Upper*, representative heat map tracings of the elevated zero maze analysis for control and socially-defeated (Stress) animals. Hot colors indicate more time spent in a specific area, while cool colors represent less time. *Lower*, quantification of elevated zero maze parameters. Distance Moved (*N* = 50 controls, 52 stress; 2-tailed; Mann-Whitney *U* = 655.5), Time in Open Field (*N* = 50 controls, 52 stress; 2-tailed; Mann-Whitney *U* = 832.5). **(C)** *Upper*, representative heat map tracings of the social interaction test. Color spectrum identical to elevated zero maze. *Lower*, quantification of social interaction test parameters. Interaction Zone Ratio (*N* = 50 controls, 52 stress; 2-tailed; Mann-Whitney *U* = 926.0), Corner Zone Ratio (*N* = 50 controls, 52 stress; 2-tailed; Mann-Whitney *U* = 1295.0).

An adapted version of the social defeat stress paradigm described by Golden et al. (2011) was utilized for all studies, and is summarized in Figure 1A. First, retired male breeder CD-1 mice (pre-screened thrice for aggressive behavior) were allowed to inhabit standard cages outfitted with two sets of food, water, and bedding 3 days before the start of an experiment to allow territory establishment by these mice. On day 1, all elements of the cage (except corncob bedding) were temporarily removed, and an experimental mouse was introduced into the cage for 5 min to allow for a physical confrontation. After the 5 min interaction period, the mice were separated within the same cage by a transparent perforated barrier, and all housing elements were placed back into the cage. The mice were then co-housed with physical separation for the remainder of the 24 h period, and the process was repeated again by rotating the experimental mouse to a different CD-1 cage for 10 days. Mice were excluded from the study if they showed signs of wounding or lameness after social defeat sessions. Control mice were pair housed using identical separation and barrier housing techniques, but not allowing for any physical confrontation between mice during the 24 h periods. At the end of the 10 day period (day 11), all mice were assessed for behavioral changes using both social interaction and elevated zero tests. After testing, control and experimental mice remained in their former co-housed barrier cage until the following day (day 12) when they were sacrificed for biological analysis.

### Elevated Zero Maze

The elevated zero maze test was utilized to assess anxiety-like behavior (Walf and Frye, 2007). An elevated circular maze consisting of 50% open and 50% enclosed quadrants was applied for these tests (50 cm diameter, 5 cm track width, 20 cm wall height, 61 cm stand height; Noldus Information Technology, Leesburg, VA, USA). Control and stress mice were introduced into a closed arm of the maze and allowed to explore the novel environment for 5 min. Runs were performed with one mouse at a time, and the maze was thoroughly cleaned using water followed by 70% ethanol (allowing time for evaporation) to eliminate olfactory variables before the next mouse was tested. Sessions were recorded, tracked, and analyzed using Noldus Ethovision XT 13 software. Tests were performed within the housing room of mice during the light cycle using approximately 265 lux of ambient lighting at the testing arena.

### Social Interaction Test

The social interaction test was utilized to assess depressive-like behavior (Golden et al., 2011). An open field chamber (40 cm wide, 40 cm long, 30 cm walls; Noldus Information Technology, Leesburg, VA, USA) was outfitted with a small wire mesh enclosure (6.5 cm wide, 10 cm long, 30 cm height; Noldus Information Technology, Leesburg, VA, USA) on one side. Control and stress mice were introduced into the open field and allowed to explore their environment with an empty mesh enclosure for 2.5 min. Runs were performed with one mouse at a time, and the chamber was thoroughly cleaned using water followed by 70% ethanol (allowing time for evaporation) to eliminate olfactory variables before the next mouse was tested. After all mice were assessed with an empty mesh enclosure, a different mesh enclosure was introduced into the open field containing a novel CD-1 aggressive mouse. Control and experimental mice were run in the aforementioned manner in the presence of an enclosed CD-1 mouse for 2.5 min. Sessions were recorded, tracked, and analyzed using Noldus Ethovision XT 13 software. Social interaction and corner zone ratios were calculated by the amount of time spent in the respective zones with a CD-1 present in the enclosure vs. absent. Tests were performed within the housing room of mice during the light cycle using approximately 265 lux of ambient lighting at the testing arena.

### T-Lymphocyte Isolation

T-lymphocytes were isolated and cultured as previously described (Case et al., 2016). Briefly, splenic T-lymphocytes were negatively selected using the EasySep Mouse T-Cell Isolation Kit (StemCell Technologies #19851, Vancouver, BC, USA). The purity (assessed by flow cytometry) and viability (assessed by a Bio-Rad TC20 Automated Cell Counter using trypan blue exclusion) of the T-lymphocytes were randomly quality controlled and found to be >90%.

### Catecholamine ELISA

Total catecholamines were assessed in plasma and splenic lysates using the 3-CAT research ELISA (Rocky Mountain Diagnostics #BAE-5600, Colorado Springs, CO, USA) as per manufacturer’s instructions. Splenic catecholamine amounts were normalized to starting splenic weights, and then to controls within respective experiments.

### Western Blot Analysis

Western blotting for the quantification of proteins was performed as previously described (Case et al., 2013). Briefly, whole-cell soluble lysate (30 μg) was separated by SDS-PAGE and transferred to a nitrocellulose membrane. Membranes were incubated with antibodies directed against tyrosine hydroxylase (TH; 1:1,000 dilution, EMD Millipore #AB152, Burlington, MA, USA) and actin (1:1,000 dilution, Sigma Aldrich #A2066, St. Louis, MO, USA) followed by horseradish peroxidase (HRP)-conjugated secondary antibodies (1:10,000, Thermo Fisher #31460, Waltham, MA, USA). Densitometric analysis of band intensity was determined using ImageJ analysis software.

### RNA Extraction, cDNA Production, and Quantitative Real-Time RT-PCR

Assessment of mRNA levels was performed as previously described (Case and Zimmerman, 2015). Briefly, total RNA was extracted from purified T-lymphocytes using the RNAeasy mini kit (Qiagen # 74104, Valencia, CA, USA) according to the manufacturer’s protocol. Concentration of RNA was determined spectrophotometrically using a Nanodrop 2000 Spectrophotometer (Thermo Fisher Scientific, Waltham, MA, USA). The High-Capacity cDNA Reverse Transcription Kit with RNase Inhibitor (Applied Biosystems #4374966, Grand Island, NY, USA) was used to obtain cDNA from total RNA. Generated cDNA was then subjected to SYBR green (Applied Biosystems #4385612, Grand Island, NY, USA) quantitative real-time PCR with primers specific to the coding sequence of the respective genes (Supplementary Table S1). PCR product specificity was determined by thermal dissociation. A threshold in the linear range of PCR amplification was selected and the cycle threshold (Ct) determined. Levels of transcripts were then normalized to the 18s rRNA loading control (ΔCT). For all analyses, 1/ΔCT was utilized to assess levels of transcripts in a directional manner relative to expression with only normalization to the 18s rRNA loading control.

### Flow Cytometric Redox Assessment

Mitochondrial-specific assessment of specific redox species was performed as previously described (Case et al., 2016). Briefly, cells were stained with 1 μM MitoSOX Red (${\text{O}}_{2}^{•-}$-sensitive mitochondrial-localized probe, Thermo Fisher Scientific #M36008, Waltham, MA, USA) for 30 min at 37°C. Cells were analyzed on a LSRII flow cytometer at 488/610 nm ex/em, and data analyzed using FlowJo software.

### Cytokine Analysis

Analysis of circulating levels of cytokines was performed using a Meso Scale Discovery 35 U-Plex Mouse Biomarker Group (#K15083K-1, Rockville, MD, USA) per manufacturer’s instructions. Samples were assessed using a Meso Scale QuickPlex SQ 120, and analyzed using Meso Scale Discovery software.

### Single-Cell RNA Sequencing

Single-cell RNA sequencing was performed on one control and one socially-defeated animal (verified by behavior testing) as a preliminary discovery method for changes in T-lymphocyte gene expression. Erythrocyte-depleted splenocyte cell suspensions were evaluated by light microscopy for debris and viability and were counted using a hemocytometer. Cell concentrations were 932 and 888 live cells/ul respectfully. Targeting approximately 2,400 single cells per sample, single cells were captured, lysed, and RNA was reverse transcribed and barcoded using a 10× Genomics Chromium instrument and Chromium Single Cell 3′ Reagent Kits v2 reagents (10× Genomics, Pleasonton, CA, USA). To construct Illumina compatible sequencing libraries the cDNA was fragmented, A-tail repaired and a double-sided bead cleanup was performed. Adapters were ligated to the cDNA fragments and the fragments were PCR amplified using unique sample index primers per manufacturer’s recommendations. Libraries were quantified by qPCR using the KAPA Library Quant Kit (Illumina) from KAPA Biosystems (Roche, Pleasonton, CA, USA). Libraries were loaded on two Illumina MidOutput V2 150 cycle flowcells at a concentration of 1.3 pM. FASTQ files were delivered to the Bioinformatics and Systems Biology Core where raw sequencing data went through 10× Genomics software cell ranger pipeline in the following order: (1) demultiplexed the Illumina sequencer’s base call files (BCLs) for each flowcell directory into FASTQ files; (2) generated single cell feature counts for each of the libraries and performed mapping/clustering; and (3) aggregated the analysis results from different libraries. A software loupe and cell ranger R kit were used for differential analyses of selected cell groups by marker gene identifications. Counts were then compared between control and stressed samples by Log2 fold changes applying two-tailed Fisher exact tests based on false discovery rate (FDR) cut-off of 0.05. The expression data was submitted to ArrayExpress repository.

### Statistics

A total of 102 animals (50 control, 52 stress) were utilized in these studies. Not all physiological parameters were able to be run on a single animal, thus, each graph is individually labeled with *N* values and statistical information utilized for a specific set of experiments. Individual data are presented along with mean ± standard error of the mean (SEM). For two group or three group comparison, significance was assessed using the Mann-Whitney *U*-test or Kruskal-Wallis test due to the non-parametric distribution of the data. Correlations were performed using linear regression with Pearson correlation coefficient calculations. Differences were considered significant at *p* < 0.05, and exact *p*-values are displayed on individual graphs.

## Results

### Repeated Social Defeat Stress Increases Anxiety-Like and Depression-Like Behavior

Several animal models exist that mimic the behavioral changes of human PTSD, and while repeated social defeat stress (Figure 1A) does not recapitulate all of these human PTSD phenotypic changes, it was chosen for this study due to its reproducible impact on inflammation (Deslauriers et al., 2018). Specific behavioral changes were first tested using an elevated zero maze to assess anxiety-like behavior and locomotor activity. Socially-defeated mice demonstrated decreased locomotor activity as evidenced by decreased total distance moved as well as increased anxiety-like behavior due to less time spent in the open arms of the elevated zero maze compared to control animals (Figure 1B). Additional parameters such as latency to first, body elongation, or head directed towards the open field only trended significance (data not shown). Depression-like and antisocial behavior was also confirmed by the use of a social interaction test, where stressed animals displayed less time spent in the interaction zone and more time in the corner zone relative to controls (Figure 1C). Together, these data support previous reports that repeated social defeat stress increases anxiety-like and depression-like behavior (Krishnan et al., 2007; Golden et al., 2011). However, these previous reports have suggested that stressed mice may be classified as “susceptible” or “resilient” based on a social interaction ratio threshold of 1.0 (Krishnan et al., 2007; Golden et al., 2011). When this analysis was performed on our mice, we observed that the “resilient” group only represented approximately 30% of the stressed mice, but moreover, was statistically different from control animals in regards to the average social interaction ratio (Supplementary Figure S1A). Furthermore, when examining locomotor activity and anxiety-like behavior on the elevated zero maze, no differences were observed between resilient and susceptible groups (Supplementary Figure S1B). Due to these discrepancies, data were processed using only major group categories (i.e., control vs. stress) as well as correlation analyses among all mice to identify dimensional individual differences among behavioral and physiological parameters.

### Increased Sympathetic Signatures Are Observed in T-Lymphocytes From Stressed Mice, but Do Not Correlate With Behavior

**Figure 2:**
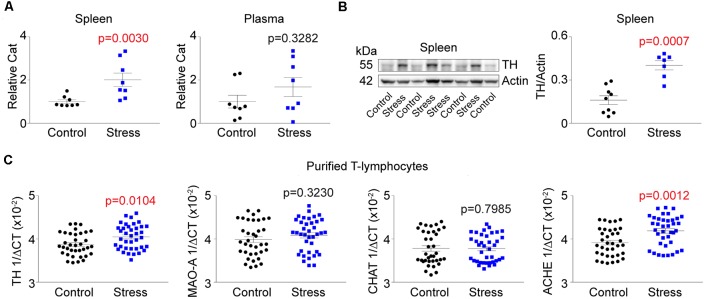
T-lymphocyte sympathetic tone is increased with psychological trauma. Plasma, whole spleens, and purified splenic T-lymphocytes were isolated following the social defeat (Stress) paradigm. **(A)** Quantification of total catecholamines (Cat) in the spleens and plasma. Splenic values were first normalized to spleen weight. All values are displayed normalized to respective controls per experiment. Spleen (*N* = 8 controls, 8 stress; 2-tailed; Mann-Whitney *U* = 5), Plasma (*N* = 8 controls, 8 stress; 2-tailed; Mann-Whitney *U* = 22). **(B)** *Left*, representative western blot analysis for splenic tyrosine hydroxylase (TH) content. *Right*, quantification of TH content by western blot. (*N* = 9 controls, 7 stress; 2-tailed, Mann-Whitney *U* = 2). **(C)** Quantitative real-time RT-PCR analysis for various neurotransmission enzyme mRNA levels in purified T-lymphocytes. Data are shown as 1/ΔCT as normalized by 18s rRNA loading control. Monoamine oxidase A (MAO-A); choline acetyltransferase (CHAT); acetylcholinesterase (ACHE). TH (*N* = 36 controls, 38 stress; 2-tailed, Mann-Whitney *U* = 448.5), MAO-A (*N* = 36 controls, 38 stress; 2-tailed, Mann-Whitney *U* = 592.0), CHAT (*N* = 36 controls, 38 stress; 2-tailed, Mann-Whitney *U* = 660), ACHE (*N* = 36 controls, 38 stress; 2-tailed, Mann-Whitney *U* = 338.5).

**Figure 3:**
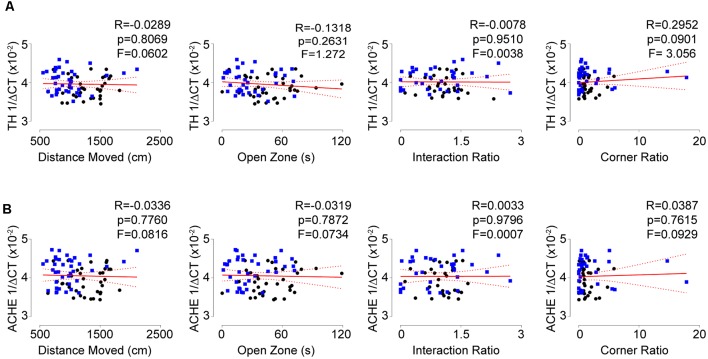
Pro-sympathetic neurotransmission signatures in splenic T-lymphocytes do not correlate with behavior.** (A)** Correlation of TH mRNA levels within splenic T-lymphocytes with anxiety-like and depression-like behavior indices. (*N* = 36 controls, 38 stress. DFn, Dfd = 1,72 for all). **(B)** Correlation of acetylcholinesterase (ACHE) mRNA levels within splenic T-lymphocytes with anxiety-like and depression-like behavior indices. (*N* = 36 controls, 38 stress. DFn, Dfd = 1,72 for all). Black circles indicate control animals; blue squares indicate socially-defeated (Stress) animals. Statistics obtained using linear regression with Pearson correlation coefficient calculations (red line; 95% confidence interval indicated as dotted red line).

Aforementioned, sympathoexcitation is a hallmark of PTSD. To assess this in our animal model, we first measured circulating levels of catecholamines at the completion of the stress induction paradigm. Unexpectedly, we did not observe any differences in circulating levels of catecholamines but identified significant increases within the spleen following social defeat (Figure 2A). This lack of increased circulating catecholamines suggested the potential for elevated sympathetic neuronal activity to the spleen. To assess this, we next evaluated the level of TH (the rate-limiting enzyme of catecholamine synthesis) protein in the spleen and observed significant increases in stressed animals (Figure 2B). To understand if the increased TH was due to potentiated neuronal expression of the protein, we excluded neurons by performing quantitative real-time RT-PCR analysis for TH mRNA in purified T-lymphocytes. To our surprise, we identified large and significant increases for TH message within T-lymphocytes (Figure 2C). We further evaluated other neurotransmitter synthetic and degradative enzyme mRNA levels within purified splenic T-lymphocytes and identified trending increases in monoamine oxidase A (MAO-A), trending decreases in choline acetyltransferase (CHAT), as well as significant increases in acetylcholinesterase (ACHE; Figure 2C). Together, these enzyme levels displayed a pro-sympathetic neurotransmission gene signature within purified T-lymphocytes after stress, which suggested the potential for lymphocyte-specific neurotransmitter production in response to stress. Interestingly, T-lymphocyte expression of genes driving a pro-sympathetic environment did not correlate with individual differences in anxiety-like or depression-like behavior (Figure 3).

### Psychological Trauma Elicits Elevations in T-Lymphocyte Mitochondrial Superoxide and Pro-inflammatory Cytokine Expression

**Figure 4:**
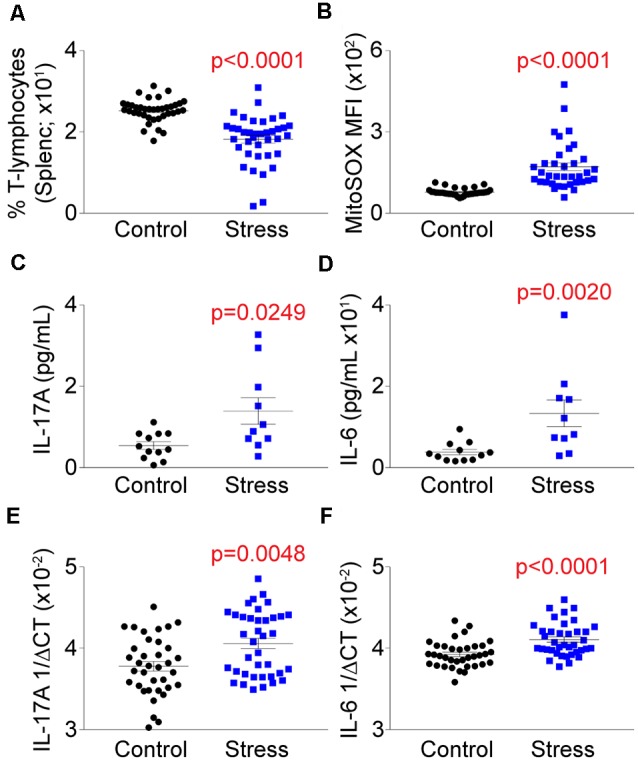
Social defeat stress elevates the mitochondrial redox and pro-inflammatory environments of splenic T-lymphocytes. Plasma, whole spleens, and purified splenic T-lymphocytes were isolated following the social defeat paradigm. **(A)** T-lymphocyte counts from control and socially-defeated (Stress) animals represented as percent of total splenocyte count. (*N* = 38 controls, 38 stress; 2-tailed; Mann-Whitney *U* = 156.5). **(B)** Quantification of MitoSOX Red mean fluorescent intensity (MFI) of splenic T-lymphocytes assessed by flow cytometry. (*N* = 35 controls, 36 stress; 2-tailed; Mann-Whitney *U* = 56). (**C,D)** Circulating levels of interleukin 17A (IL-17A) and interleukin 6 (IL-6) assessed by mesoscale multiplex analysis. IL-17A (*N* = 12 controls, 10 stress, 2-tailed; Mann-Whitney *U* = 26.0), IL-6 (*N* = 12 controls, 10 stress, 2-tailed; Mann-Whitney *U* = 15.0). (**E,F)** Quantitative real-time RT-PCR analysis of IL-17A and IL-6 mRNA within splenic T-lymphocytes. Data are shown as 1/ΔCT as normalized by 18s rRNA loading control. (*N* = 36 controls, 38 stress; 2-tailed; Mann-Whitney *U* = 425.5), IL-6 (*N* = 36 controls, 38 stress; 2-tailed; Mann-Whitney *U* = 330.5).

Our previous work demonstrated that T-lymphocytes exposed to NE expressed increased levels of IL-6 and IL-17A that was driven in part due to amplified mitochondrial superoxide production (Case et al., 2016). Understanding that social defeat elevated catecholamine levels in proximity to T-lymphocytes, we examined the effects of stress on T-lymphocyte redox and inflammatory environments. We first observed that social defeat caused a significant decrease in the percentage of splenic T-lymphocytes (Figure 4A), which has been previously reported in a similar trauma animal model (Avitsur et al., 2002). Assessment of these remaining splenic T-lymphocytes showed an approximate 2-fold induction of mitochondrial superoxide levels compared to controls (Figure 4B). Mitochondrial superoxide was not altered in circulating T-lymphocytes (data not shown), which further supported the importance of direct interaction with catecholamines. No change was observed in splenic T-lymphocyte nitric oxide levels from socially-defeated animals (Supplementary Figure S2), demonstrating not all redox signaling is perturbed with psychological stress. IL-6 and IL-17A levels were increased in circulation of socially-defeated animals (Figures 4C,D), and mRNA levels for these cytokines were also specifically and significantly elevated within splenic T-lymphocytes (Figures 4E,F). Overall, these data confirm and extend our previous *in vitro* findings in a relevant *in vivo* model of psychological trauma that catecholamines impact T-lymphocyte inflammation likely *via* redox mechanisms.

### T-Lymphocyte Mitochondrial Superoxide Correlates With Anxiety-Like Behavior, While IL-6 Expression Associates With Depression-Like Behavior

**Figure 5:**
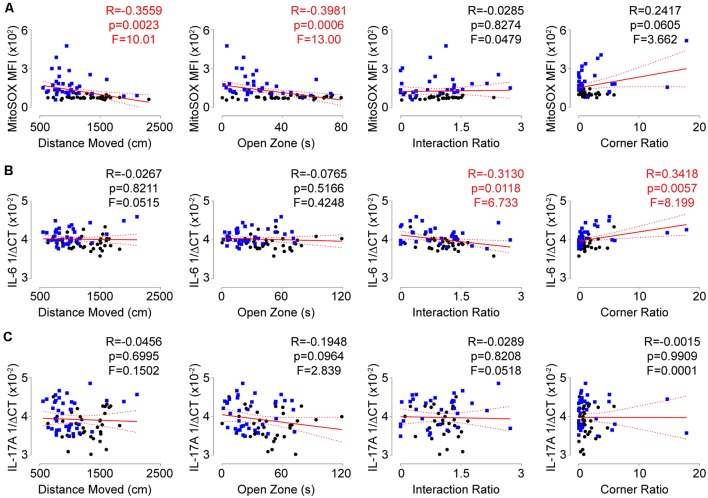
Redox and inflammatory parameters show differential associations with behavior. **(A)** Correlation of splenic T-lymphocyte MitoSOX Red MFI with anxiety-like and depression-like behavior indices. (*N* = 35 controls, 36 stress. DFn, Dfd = 1,69 for all). **(B)** Correlation of splenic T-lymphocyte IL-6 mRNA levels with anxiety-like and depression-like behavior indices. (*N* = 36 controls, 38 stress. DFn, Dfd = 1,72 for all). **(C)** Correlation of splenic T-lymphocyte IL-17A mRNA levels with anxiety-like and depression-like behavior indices. (*N* = 36 controls, 38 stress. DFn, Dfd = 1,72 for all). Black circles indicate control animals; blue squares indicate socially-defeated (Stress) animals. Statistics obtained using linear regression with Pearson correlation coefficient calculations (red line; 95% confidence interval indicated as dotted red line). Values highlighted in red demonstrate statistical significance.

To assess if the T-lymphocyte mitochondrial redox and inflammatory environments had any impact on behavior, we performed correlation analyses on all animals comparing individual behavioral indices and these physiological readouts. Intriguingly, a positive correlation was observed between T-lymphocyte mitochondrial superoxide levels and anxiety-like behavior, but not depression-like behavior (Figure 5A). In contrast, splenic T-lymphocyte expression of IL-6 or IL-17A did not correlate with anxiety-like behavior indices but IL-6 positively correlated with depression-like behavior indices (Figures 5B,C), which has been previously reported (Hodes et al., 2014). Together, these data suggest that splenic T-lymphocyte mitochondrial superoxide levels may serve as an indicator of anxiety-like behavior, whereas specific inflammatory components may be more predictive of depressive-like symptoms in the social defeat stress model.

### Significant Associations Exist Between the Autonomic, Redox, and Inflammatory Signatures of T-Lymphocytes

**Figure 6:**
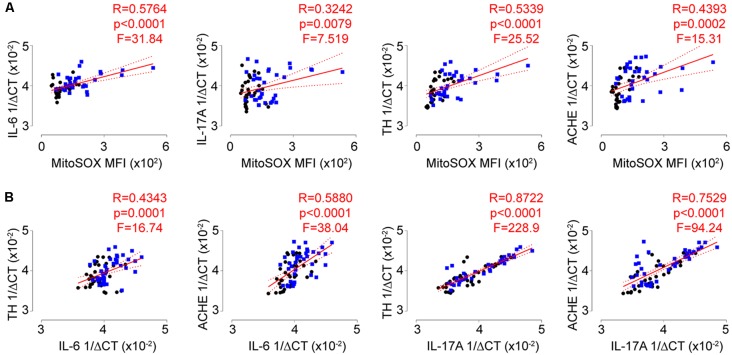
Autonomic, redox, and inflammatory T-lymphocyte signatures are highly correlated. **(A)** Correlation of splenic T-lymphocyte MitoSOX Red MFI with splenic T-lymphocyte inflammatory (interleukin 6, IL-6; interleukin 17A, IL-17A) and autonomic (tyrosine hydroxylase, TH; acetylcholinesterase, ACHE) genes.** (***N* = 32 controls, 34 stress. DFn, Dfd = 1,64 for all). **(B)** Correlation of splenic T-lymphocyte inflammatory (IL-6; IL-17A) with autonomic (TH; ACHE) genes. (*N* = 36 controls, 38 stress. DFn, Dfd = 1,72 for all). Black circles indicate control animals; blue squares indicate socially-defeated (Stress) animals. Statistics obtained using linear regression with Pearson correlation coefficient calculations (red line; 95% confidence interval indicated as dotted red line). Values highlighted in red demonstrate statistical significance.

While the biological changes observed with repeated social defeat did not completely associate with individual behavioral phenotypes, we next set out to address if these physiological changes correlated with each other. Strikingly, we observed strong and significant positive correlations among all combinations of biological measures including T-lymphocyte mitochondrial superoxide and IL-6, IL-17A, TH, and ACHE transcript levels (Figures 6A,B; Supplementary Figures S3A,B). These data are highly suggestive of crosstalk between the autonomic, redox, and inflammatory pathways in T-lymphocytes during psychological trauma.

### Identification of Calprotectin as a Novel Indicator of Behavioral and Physiological Changes During Psychological Trauma

**Figure 7:**
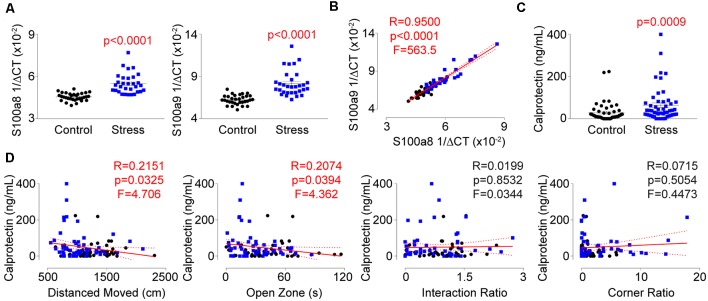
Identification of calprotectin as a novel marker of psychological trauma. Plasma, whole spleens, and purified splenic T-lymphocytes were isolated following the social defeat paradigm. **(A)** Quantitative real-time RT-PCR analysis of S100a8 and S100a9 mRNA levels within splenic T-lymphocytes. Data are shown as 1/ΔCT as normalized by 18s rRNA loading control. S100a8 (*N* = 32 controls, 31 stress; 2-tailed; Mann-Whitney *U* = 58.0), S100a9 (*N* = 32 controls, 31 stress; 2-tailed; Mann-Whitney *U* = 47.5). **(B)** Correlation of splenic T-lymphocyte levels of S100a8 and S100a9 mRNA levels. (*N* = 32 controls, 31 stress. DFn, Dfd = 1,61). Statistics obtained using linear regression with Pearson correlation coefficient calculations (red line; 95% confidence interval indicated as dotted red line). **(C)** Circulating calprotectin levels assessed by ELISA. (*N* = 50 controls, 52 stress; 2-tailed, Mann-Whitney *U* = 756). **(D)** Correlation of circulating calprotectin levels with anxiety-like and depression-like behavior indices. (*N* = 50 controls, 52 stress. DFn, Dfd = 1,100 for all). Black circles indicate control animals; blue squares indicate socially-defeated (Stress) animals. Statistics obtained using linear regression with Pearson correlation coefficient calculations (red line; 95% confidence interval indicated as dotted red line). Values highlighted in red demonstrate statistical significance.

Understanding that repeated social defeat stress significantly impacted the expression of several genes within T-lymphocytes, we next performed single-cell RNA sequencing analysis on splenocytes from socially-defeated and control animals (Supplementary Table S2). Interestingly, two of the most significantly upregulated genes in the T-lymphocyte population were calgranulin A (S100a8; Log2 fold increase +4.41, *p* = 8.6 × 10^−19^) and calgranulin B (S100a9; Log2 fold increase 4.28, *p* = 1.3 × 10^−13^), which together form the heterodimeric protein calprotectin. Calprotectin possesses both intracellular and extracellular properties but is thought to be primarily produced by neutrophils and monocytes making its detection in T-lymphocytes quite unexpected. We validated mRNA levels of the respective transcripts and observed large inductions within splenic T-lymphocytes from stressed animals that correlated strongly with one another (Figures 7A,B). Calprotectin protein was also increased approximately 3-fold in circulation of stressed animals and positively correlated only with anxiety-like behavior (Figures 7C,D). T-lymphocyte expression of S100a8 and S100a9 showed a similar positive correlation with anxiety-like behavior, but also positively correlated with the corner zone ratio of the social interaction test (Supplementary Figures S4A,B). Interestingly, S100a8 and S100a9 mRNA levels in splenic T-lymphocytes only correlated with mitochondrial superoxide levels, IL-6, and ACHE expression, suggesting a dissociative expression pattern compared to the other dysregulated genes (Supplementary Figures S5A,B). Together, we describe for the first time the observation of psychological trauma-induced calprotectin expression that associates with both behavioral and physiological alterations of repeated social defeat stress.

## Discussion

In the current study, we identify several previously undescribed links between the autonomic, redox, and inflammatory environments of splenic T-lymphocytes during psychological trauma. Together, these provide new insights into the regulatory control of the adaptive immune system during stress and suggest potential pathways which may lead to the increased incidence of inflammatory comorbidities in diseases like PTSD.

PTSD is a multifaceted disease that has proven difficult to replicate in animal models. A recent review by Deslauriers et al. (2018) has elegantly summarized accepted animal models that recapitulate PTSD behavioral and biological phenotypes. There are currently six animal models that are able to mimic the behavioral and biological phenotypes of PTSD, but each has demonstrated at least one phenotype of the human disease that is not able to be fully recapitulated. We chose the repeated social defeat model based on its ability to robustly and consistently produce increased anxiety-like behavior, depression-like behavior, as well as peripheral inflammation (Deslauriers et al., 2018). As described in this review article, repeated social defeat is only one of two accepted animal models that produce a peripheral inflammatory response similar to human PTSD, which was the primary focus of this work. However, social defeat is limited in the fact that it does not demonstrate hallmark phenotypes of PTSD such as decreased fear extinction or increased HPA feedback. Additionally, the standard repeated social defeat does not allow for the use of female mice, which precludes examination of sex differences [An alternative version of social defeat using genetically modified CD-1 mice has been reported to be used with females (Takahashi et al., 2017)]. Therefore, while we have identified novel redox and inflammatory findings using a repeated social defeat model, due to the limitations of this model, further investigation is warranted to validate these observations in additional models of PTSD such as unpredictable variable stress, predator exposure, inescapable foot shocks, or single prolonged stress.

Repeated social defeat has also previously been reported to generate both “resilient” and “susceptible” phenotypes across stressed animals (Krishnan et al., 2007; Golden et al., 2011; Friedman et al., 2014). This grouping is based off of the social interaction behavior test, and the threshold cut-off is set at 1.0 (the value in which a mouse will enter the interaction zone at the same frequency with and without a CD-1 present). Using these categories, these two groups of mice have been shown to have different physiological phenotypes (Krishnan et al., 2007; Friedman et al., 2014; Hodes et al., 2014), but in contrast, also show no differences in many behavioral phenotypes (Krishnan et al., 2007). When performing this categorization on our animals, we found that approximately 30% of stressed animals were found to be “resilient,” which is within the range (albeit the extreme low end) previously reported by Golden et al. (2011). However, unlike previous reports, the resilient group here was statistically different in regards to their social interaction ratio (and other parameters) compared to control animals. When examining the data closely, this difference is because animals demonstrating low social interaction ratios exist among control animals, but yet are all averaged as one composite group overlooking this natural variation. Therefore, we have pursued an alternative approach examining both group statistics (i.e., control vs. stress), and also individual statistics across dimensions of behavior and physiology. By using this type of analysis, we aim to mirror the National Institutes of Mental Health’s Research Domain Criteria (RDoC) method that attempts to limit categorization diagnoses but instead examine individuals across various dimensions of behavior. In doing so, we find that correlations hold true across all animals dependent upon behavior phenotype, not trauma exposure, which we believe may be more reflective of the human condition.

One of the first intriguing findings of this work is that we show that T-lymphocytes express their own neurotransmission synthetic and degradative machinery that is dysregulated during psychological stress, suggesting the potential for T-lymphocyte-driven microenvironmental control of inflammation. Indeed, expression of these pro-sympathetic genes was tightly correlated with both mitochondrial superoxide and pro-inflammatory cytokine levels within T-lymphocytes, further supporting this pro-inflammatory hypothesis. However, T-lymphocyte expression of neurotransmission enzymes is a relatively new observation, and little is known regarding the contribution of these signaling pathways in these adaptive immune systems. TH expression and endogenous catecholamine production by T-lymphocytes was first observed in the mid-1990s and its function was shown to suppress lymphocyte proliferation and differentiation, which was suggested as a possible negative feedback mechanism to attenuate inflammation (Bergquist et al., 1994). Work from Yu-Ping Peng and Yi-Hua Qiu have also shown an overall suppressive phenotype of TH expression within T-lymphocytes. Several studies from this group have shown that TH expression and T-lymphocyte catecholamine production leads to a pro-T_H_2 phenotype with suppression of T_H_1 (Qiu et al., 2004, 2005; Liu et al., 2012). They have more recently demonstrated that TH expression correlates with the pro-inflammatory T_H_17 subtype of T-lymphocytes, however, forced over-expression of TH in T-lymphocytes suppressed the polarization to the T_H_17 phenotype (Wang et al., 2016). This data again suggests that T-lymphocytes may upregulate TH in pro-inflammatory subtypes as an auto-regulatory feedback mechanism to control inflammation. However, contradicting results exist showing both positive and negative effects of endogenous catecholamine production on anti-inflammatory regulatory T-lymphocyte function (Cosentino et al., 2007; Wang et al., 2016). Here, we demonstrate a significant elevation of TH expression within T-lymphocytes after repeated psychological trauma. Given the high correlation between TH and pro-inflammation gene expression levels among T-lymphocytes, these data support that either TH promotes inflammation within T-lymphocytes, or is upregulated in a compensatory manner to counteract the pro-inflammatory phenotype. Interestingly, T-lymphocyte TH levels did not correlate with behavioral changes after stress-induction, suggesting psychological manifestations after stress may not be directly coupled to autonomic changes.

The cholinergic system has also shown significant regulatory control over T-lymphocytes, yet, lymphoid organs such as the spleen are not cholinergically innervated (Dale and Dudley, 1929; Nance and Sanders, 2007). Acetylcholine, CHAT, and ACHE are all endogenously produced within T-lymphocytes (Szelényi et al., 1982; Rinner and Schauenstein, 1993; Rinner et al., 1998; Kawashima et al., 2007), and the regulation of acetylcholine on T-lymphocytes is complex, extensive, and has been previously reviewed (Fujii et al., 2017). Overall, due to the spleen being exclusively innervated by the sympathetic splenic nerve, it is believed the primary source of acetylcholine in this organ is CHAT-expressing T-lymphocytes (Pavlov and Tracey, 2005; Rosas-Ballina et al., 2011). Because of this, much work has focused on the role of CHAT-positive T-lymphocytes, while those expressing ACHE have been relatively understudied. Similar to CHAT, ACHE is significantly upregulated during T-lymphocyte activation suggesting a critical regulatory role in the modulation of cellular function (Szelényi et al., 1987). Additionally, pharmacological inhibition of ACHE in T-lymphocytes reduced the production of IL-17A, suggesting the degradation of acetylcholine by ACHE may be important in the pro-inflammatory response (Nizri et al., 2008). Here, we also demonstrate a significant induction of ACHE mRNA in splenic T-lymphocytes after stress, which like TH, correlated significantly with pro-inflammatory cytokine expression and mitochondrial superoxide levels in these cells. Together, these data suggest the potential for a causal relationship between sympathetic autonomic balance and the inflammatory phenotype of T-lymphocytes, but further studies are needed to identify the mechanistic nature of these neurotransmission enzymes during psychological trauma.

While our work demonstrated the effects of psychological stress on T-lymphocytes, it is interesting to note that previous work has shown that peripheral T-lymphocytes may conversely impact behavior. Original studies utilized immunodeficient mice and could demonstrate that reconstitution with naïve T-lymphocytes restored enhanced cognitive function (Kipnis et al., 2004; Ziv et al., 2006). Anxiety-like and depression-like behavior have also been improved by the addition of naïve T-lymphocytes to immunodeficient animals (Cohen et al., 2006; Lewitus et al., 2008, 2009), whereas pro-inflammatory T-lymphocytes potentiate pathological behavior (Beurel et al., 2013). However, behavioral changes in response to T-lymphocytes do not appear to be universal and vary among stress-induction paradigms (Clark et al., 2014). Here, we demonstrate that repeated social defeat stress increases pro-inflammatory gene expression within T-lymphocytes, however, expression of these cytokines only associated with depression-like and not anxiety-like behavior. Correlations between circulating IL-6 levels and depression-like behavior have been previously observed when categorizing socially-defeated animals into susceptible and resilient groups (Hodes et al., 2014), but our data suggest individual differences display more as a spectrum as opposed to two separate entities. Additional studies are needed to identify if these correlations are specific to the social defeat paradigm, or may be more broadly applied.

Herein, we additionally identified two previously undescribed observations. First, we elucidate that repeated social defeat stress significantly increases mitochondrial superoxide levels within T-lymphocytes. This phenomenon appears specific to splenic T-lymphocytes, in that other cells of the spleen or even T-lymphocytes in circulation did not alter their mitochondrial redox environments after stress (data not shown). We previously observed that exogenous NE applied to T-lymphocytes *ex vivo* could increase mitochondrial superoxide levels, which appeared to regulate both IL-6 and IL-17A expression in these cells (Case et al., 2016). We posit that the elevated catecholamine levels in the spleens of social defeat animals may be eliciting a similar effect. Additionally, the highly significant positive correlations between mitochondrial superoxide and IL-6 or IL-17A levels with repeated social defeat is suggestive of similar mechanisms at play as well. The underlying cause of the increase in mitochondrial superoxide is currently unknown, but it is hypothesized that this induction may be occurring due to a potentiated metabolic state of the T-lymphocytes that occurs during activation (Pearce et al., 2013). However, our previous work would suggest that mitochondrial superoxide plays a critical regulatory role in T-lymphocyte activation and differentiation, and is not simply a by-product of another cellular process (Case et al., 2016). Another potential source of superoxide within T-lymphocytes could be from the direct oxidation of catecholamines. Utilizing a mouse model of NE infusion along with a combination of adrenergic receptor and catecholamine transport inhibitors, we previously identified that direct intracellular oxidation of catecholamines was not the primary source of superoxide within T-lymphocytes (Case and Zimmerman, 2015), but this possibility has not been exhaustively tested yet in our psychological trauma model. Last, we observed that T-lymphocyte-specific mitochondrial superoxide levels positively correlated with anxiety-like behavior after social defeat stress. Taken together with the tight positive correlations among mitochondrial superoxide levels, pro-inflammatory cytokine expression, and neurotransmission gene expression in T-lymphocytes, our findings suggest the mitochondrial redox environment of these cells may be causally involved in the pro-inflammatory nature of these cells, which could potentiate pathological anxiety-like behavior similar to what is observed in PTSD.

The additional novel observation described here was the identification of elevated calprotectin levels in socially-defeated animals. Aforementioned, calprotectin is a heterodimer of two proteins: calgranulin A and calgranulin B (encoded by S100a8 and S100a9, respectively). Calprotectin primarily serves as an extracellular protein where its known antimicrobial function is the sequestration of metals such as iron, manganese, and zinc, and is mainly produced by neutrophils and monocytes (Striz and Trebichavsky, 2004). Thus, our observation of stress-regulated S100a8 and S100a9 mRNA expression in T-lymphocytes was highly unexpected. In addition to its antimicrobial functions, calprotectin also serves as a damage-associated molecular pattern (DAMP) to activate other immune cells and promote inflammation (Vogl et al., 2007; Foell et al., 2008; Ehrchen et al., 2009). In fact, calprotectin binding to toll-like receptor 4 (TLR4) on the surface of T-lymphocytes enhances the IL-17A production from these cells, and this ability is attenuated in cells lacking TLR4 (Loser et al., 2010). While we did not observe a correlation with T-lymphocyte expressed calprotectin and IL-17A, we did observe a positive correlation between T-lymphocyte IL-17A and circulating calprotectin (data not shown). Interestingly, we observed correlations between calprotectin and IL-6 mRNA levels in T-lymphocytes, but not between IL-6 and circulating calprotectin. This is suggestive of different intracellular and extracellular functions of calprotectin that may be regulating T-lymphocyte inflammation. Additionally, calprotectin has previously been shown to be redox-regulated within its canonically-expressed cell types, and this protein plays a direct role in intracellular redox signaling (Jia et al., 2014). We observed that calprotectin expression correlated with mitochondrial superoxide levels in T-lymphocytes, which further suggests this protein may be a previously undescribed regulatory player in T-lymphocyte redox signaling. Last, calprotectin (in T-lymphocytes and in circulation) correlated with anxiety-like behavior. The downstream ramifications of this observation are unclear but suggest that calprotectin may serve as a biological marker for this specific behavioral manifestation. Moreover, knowing that calprotectin has a functional role in promoting inflammation in autoimmune diseases, the presence of this protein may also suggest a predisposition to the development of comorbid inflammatory diseases after trauma. Taken together, this research opens a new avenue of investigation into the mechanistic roles of neurotransmission, inflammation, and redox into the long-term consequences of psychological traumatic diseases like PTSD.

## Ethics Statement

This study was carried out in accordance with the recommendations of the University of Nebraska Medical Center Institutional Animal Care and Use Committee. The protocol was approved by the University of Nebraska Medical Center Institutional Animal Care and Use Committee.

## Author Contributions

CM, SE, CC and AC designed research studies. CM, SE, CC, AK and AC conducted experiments and acquired, analyzed data. AC provided experimental oversight and wrote the manuscript.

## Conflict of Interest Statement

The authors declare that the research was conducted in the absence of any commercial or financial relationships that could be construed as a potential conflict of interest.
