# Supplementary material for: Autonomic and Redox Imbalance Correlates With T-Lymphocyte Inflammation in a Model of Chronic Social Defeat Stress
Source: Front Behav Neurosci. 2019 May 14;13:103. doi: 10.3389/fnbeh.2019.00103 (PMC6527882; doi:10.3389/fnbeh.2019.00103)
Supplement: Supplementary file 1 [file Data_Sheet_1.pdf]

Supplemental Data

**Oligo Name**	**Sequence (5' to 3')**
18s F	GCCCGAAGCGTTTACTTTGA
18s R	TCATGGCCTCAGTTCCGAA
TH F	TCACTTCTTGAAGGAACGGACT
TH R	CACGGGCAGACAGTAGACC
MAO-A F	CGGATATTCTCAGTCACCAATG
MAO-A R	ATTTGGCCAGAGCCACCTA
CHAT F	GGTTCGGTGCGTAACAGC
CHAT R	GCGATTCTTAATCCAGAGTAGCA
ACHE F	CCCTCGCTGAACTACACCAC
ACHE R	TTGGAGTCTCGAGGGTCATT
IL-6 F	GCTACCAAACTGGATATAATCAGGA
IL-6 R	CCAGGTAGCTATGGTACTCCAGAA
IL-17A F	CAGGGAGAGCTTCATCTGTGT
IL-17A R	GCTGAGCTTTGAGGGATGAT
S100a8 F	TCCTTGCGATGGTGATAAAA
S100a8 R	GGCCAGAAGCTCTGCTACTC
S100a9 F	GACACCCTGACACCCTGAG
S100a9 R	TGAGGGCTTCATTTCTCTTCTC

Supplemental Table 1. Mouse-specific real-time RT-PCR primer sequences.

EnsemblID	GeneName	Log2 Fold Change (Stress vs Control)	p Value
ENSMUSG00000056032	BC018473	7.93	7.99E-25
ENSMUSG00000056054	S100a8	4.41	8.60E-19
ENSMUSG00000038357	Camp	5.89	1.67E-18
ENSMUSG00000056071	S100a9	4.25	1.28E-13
ENSMUSG00000094724	Rnaset2b	-4.59	1.57E-11
ENSMUSG00000095079	Igha	4.76	1.28E-09
ENSMUSG00000032484	Ngp	4.93	4.78E-09
ENSMUSG00000026822	Lcn2	3.83	0.000472002
ENSMUSG00000008348	Ubc	-1.85	0.001449797
ENSMUSG00000057286	St6galnac2	4.83	0.002718216
ENSMUSG00000045193	Cirbp	-1.96	0.012717186
ENSMUSG00000052031	Tagap1	2.64	0.024612402
ENSMUSG00000051748	Wfdc21	3.72	0.042875559

Supplemental Table 2. Significant T-lymphocyte gene changes by single cell RNA sequencing.

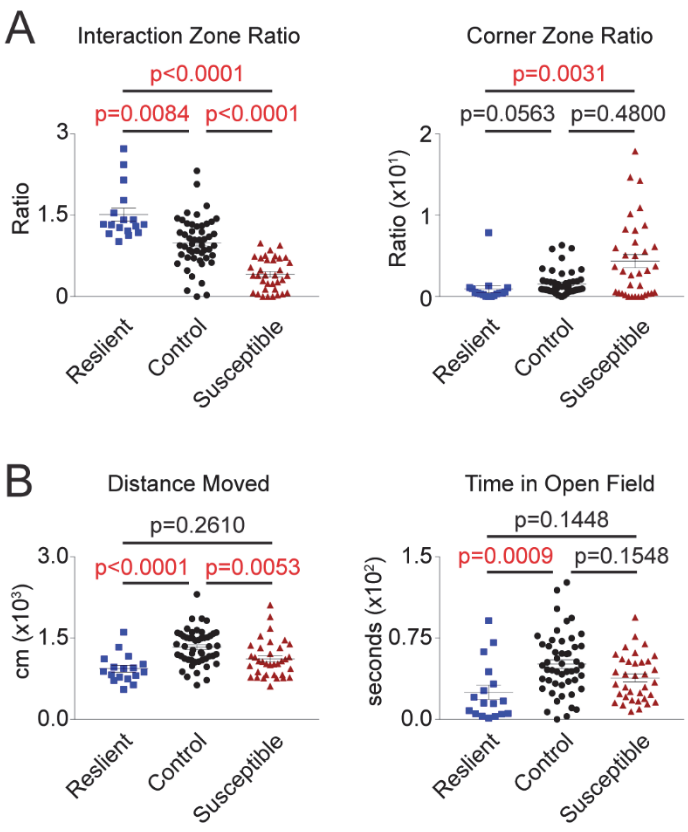

Supplemental Figure 1. Social interaction-based categorization does not apply to other behavioral phenotypes. A. Quantification of social interaction test parameters. Stress animals were separated into "resilient" or "susceptible" categories based on a social interaction ratio of either >1 or <1, respectively. Social Interaction Zone (N=17 resilient, 50 controls, 35 susceptible; 3 comparisons; α=0.05; Kruskal-Wallis=51.25), Corner Zone Ratio (N=17 resilient, 50 controls, 35 susceptible; 3 comparisons; α=0.05; Kruskal-Wallis=10.76). **B**. Quantification of elevated zero maze parameters. Distance Moved (N=17 resilient, 50 controls, 35 susceptible; 3 comparisons; α=0.05; Kruskal-Wallis=21.54), Time in Open Field (N=17 resilient, 50 controls, 35 susceptible; 3 comparisons; α=0.05; Kruskal-Wallis=13.69).

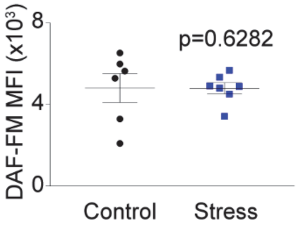

Supplemental Figure 2. T-lymphocyte nitric oxide levels are unchanged with social defeat stress. Quantification of DAF-FM mean fluorescent intensity (MFI) of splenic T-lymphocytes assessed by flow cytometry. (N=6 controls, 7 stress; 2-tailed; Mann-Whitney U=17.0).

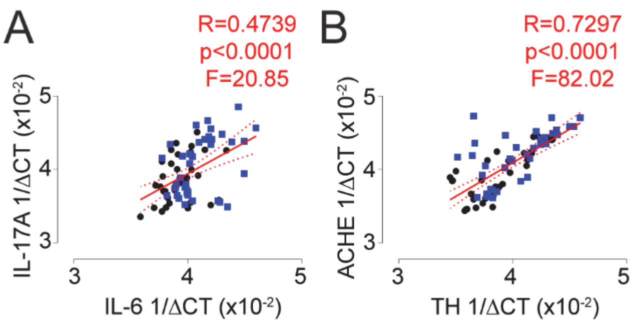

Supplemental Figure 3. Positive correlations exist between inflammatory and autonomic signatures in splenic T-lymphocytes. A. Correlation of splenic T-lymphocyte interleukin 6 (IL-6) and interleukin 17A (IL-17A) mRNA levels. (N=36 controls, 38 stress. DFn, Dfd= 1,72). **B**. Correlation of splenic T-lymphocyte tyrosine hydroxylase (TH) and acetylcholinesterase (ACHE) mRNA levels. (N=36 controls, 38 stress. DFn, Dfd= 1,72). Black circles indicate control animals; blue squares indicate socially-defeated (Stress) animals. Statistics obtained using linear regression with Pearson correlation coefficient calculations (red line; 95% confidence interval indicated as dotted red line). Values highlighted in red demonstrate statistical significance.

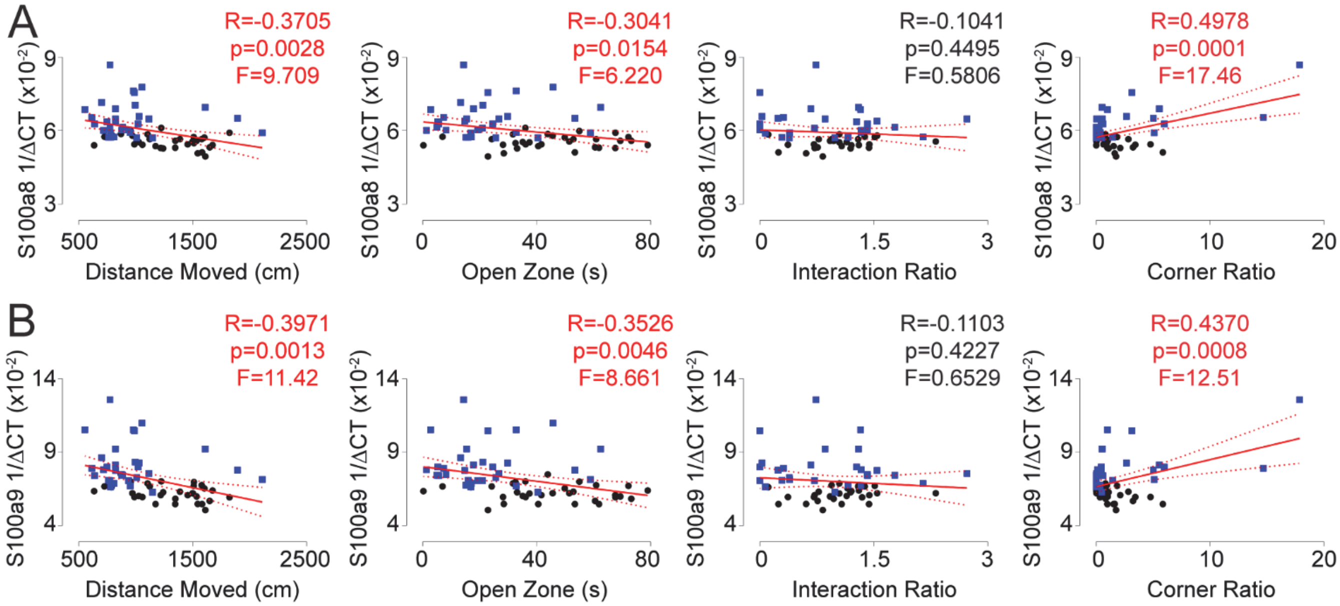

Supplemental Figure 4. Calprotectin mRNA levels positively correlate with stress-related behavior changes. A. Correlation of splenic T-lymphocyte S100a8 mRNA levels with anxiety-like and depression-like behavior indices. (N=32 controls, 31 stress. DFn, Dfd= 1,61 for all). **B.** Correlation of splenic T-lymphocyte S100a9 mRNA levels with anxiety-like and depression-like behavior indices. (N=32 controls, 31 stress. DFn, Dfd= 1,61 for all). Black circles indicate control animals; blue squares indicate socially-defeated (Stress) animals. Statistics obtained using linear regression with Pearson correlation coefficient calculations (red line; 95% confidence interval indicated as dotted red line). Values highlighted in red demonstrate statistical significance.

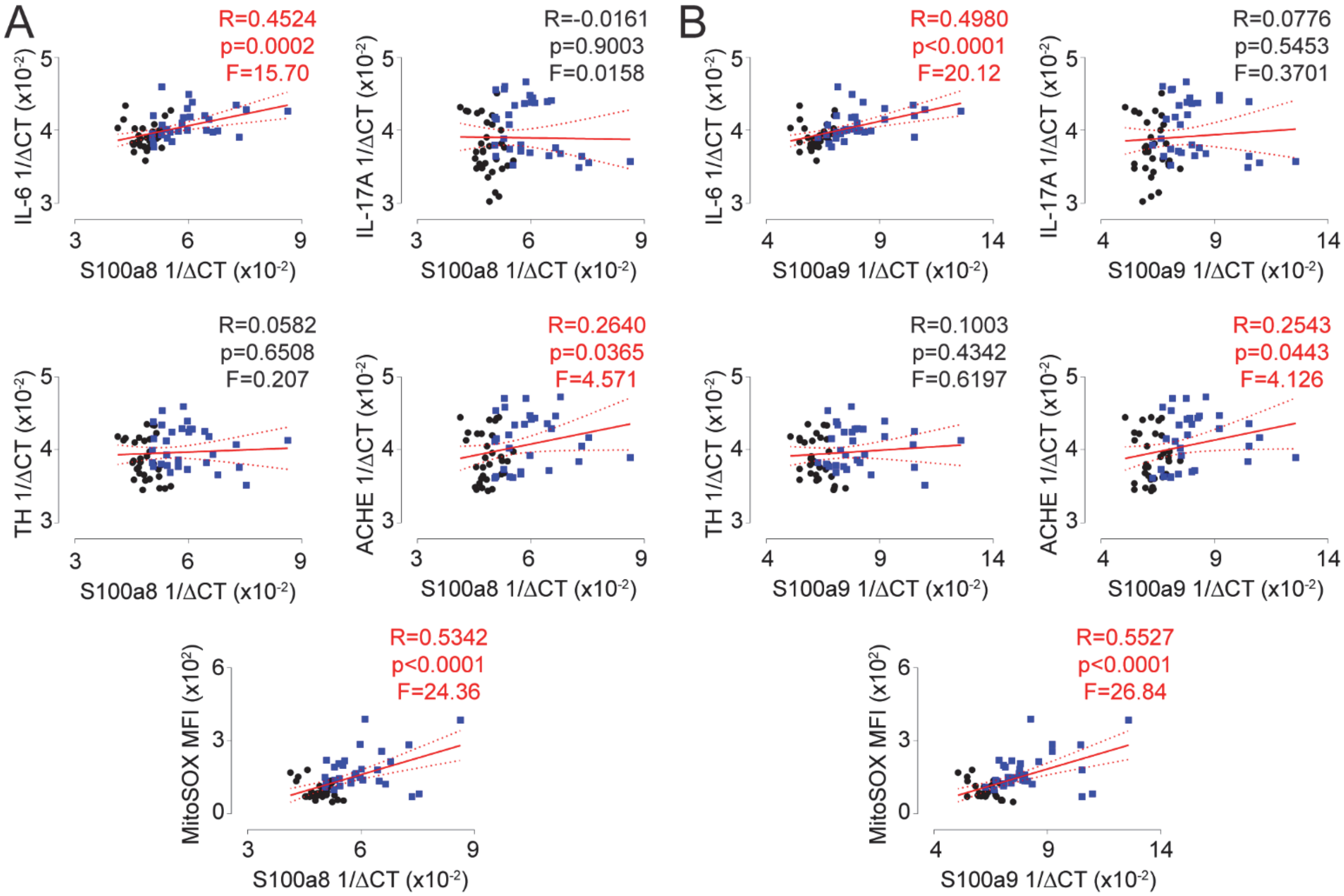

Supplemental Figure 5. Calprotectin associates with only interleukin 6 (IL-6) and mitochondrial superoxide within splenic T-lymphocytes. A. Correlation of splenic T-lymphocyte S100a8 mRNA levels with splenic T-lymphocyte inflammatory (interleukin 6, IL-6; interleukin 17A, IL-17A) genes, autonomic (tyrosine hydroxylase, TH; acetylcholinesterase, ACHE) genes, and MitoSOX Red mean fluorescent intensity (MFI). (N=32 controls, 31 stress. DFn, Dfd= 1,61 for all). **B.** Correlation of splenic T-lymphocyte S100a9 mRNA levels with splenic T-lymphocyte inflammatory (interleukin 6, IL-6; interleukin 17A, IL-17A) genes, autonomic (tyrosine hydroxylase, TH; acetylcholinesterase, ACHE) genes, and MitoSOX Red mean fluorescent intensity (MFI). (N=32 controls, 31 stress. DFn, Dfd= 1,61 for all). Black circles indicate control animals; blue squares indicate socially-defeated (Stress) animals. Statistics obtained using linear regression with Pearson correlation coefficient calculations (red line; 95% confidence interval indicated as dotted red line). Values highlighted in red demonstrate statistical significance.
